# Registered nurses' perceptions of their career—An interview study

**DOI:** 10.1111/jonm.13796

**Published:** 2022-09-16

**Authors:** Hanna Kallio, Mari Kangasniemi, Marja Hult

**Affiliations:** ^1^ Department of Nursing Science University of Turku Turku Finland; ^2^ Department of Nursing Science University of Eastern Finland Kuopio Finland

**Keywords:** career, qualitative research, registered nurses, working life

## Abstract

**Aim:**

We aim to explore registered nurses' perceptions of their career.

**Background:**

Career development options have been found to increase attraction to nursing and support nurses' engagement with their organization and profession.

**Methods:**

We collected qualitative individual interviews with 23 registered nurses; data were analysed with thematic analysis and reported according to the consolidated criteria for reporting qualitative research (COREQ) criteria.

**Results:**

Three themes emerged: career choices, career engagement and career development. Participants had chosen a nursing career because they perceived it as humane, people oriented, meaningful, diverse and secure work. Participants' engagement in their career was connected to the content of the work, in which direct patient care was central. Nurses connected career development with high competency, independence, influence and meaningful working life experience. However, they perceived career development opportunities as minute within direct patient care.

**Conclusions:**

Career development opportunities for nurses in direct patient care are needed to foster their career engagement and the attractiveness of the nursing profession. Further research is needed on the career planning and development of nurses working in patient care.

**Implications for Nursing Management:**

Nurse managers must play a central role in engaging nurses in their careers and promoting their competency and career planning and development in organizations.

## 1 BACKGROUND

The escalating worldwide nursing shortage (International Council of Nurses, 2021) is the major challenge for nurse leaders and managers. There is an urgent need to develop both nurses' working life and the public image of nursing as a career, as it reflects on its attractiveness (Glerean et al., 2017) and hence influences the availability of labour (Bayliss‐Pratt et al., 2020). Societal trends, such as population ageing and climate change, increase noncommunicable diseases and therefore the need for care and a nursing workforce to provide such care. However, several deficiencies have been recognized that hinder nurses' motivation to engage in their career: excessive workload (Kox et al., 2020) and underpay (Bayliss‐Pratt et al., 2020) in particular.

Nursing has been a traditional ‘calling profession’, often referred to altruistic and sacrificing image of a nurse as an unambitious subordinate, thus conflicting with career pursuits (Meleis, 1997). However, previous research has shown that high competency and training opportunities are important for nurses' experience of meaningful careers and ability to provide high‐quality care (Hariyati et al., 2017) in a burdensome, constantly changing health care environment (Price & Reichert, 2017). Research has also addressed the fact that nurses value autonomy in their work (Rakhab et al., 2021) and that, as they work closely with patients, they play a vital role in developing care practices (Kallio et al., 2018). Furthermore, it is notable that younger generations have been shown more likely to base their career choices on financial comfort rather than living a calling with poor pay (Carter, 2014).

Opportunities for career development have been recognized to resonate with nurses' experience of a meaningful working life (Moore et al., 2019) and to be one of the key factors in workforce retention (Brook et al., 2019; Marufu et al., 2021; Yarbrough et al., 2017). However, factors supporting career development (Eley et al., 2012; Marufu et al., 2021), such as clear progression routes (Rakhab et al., 2021) and advocacy by leadership (Price & Reichert, 2017), have often been found lacking. Research describing registered nurses' perceptions of their careers is scarce. We aimed to provide such knowledge. This knowledge will be beneficial for nurse managers to support nurses in their career planning and is needed from the perspective of competence management and future competence needs.

## 2 METHODS

### 2.1 Study design, participants and recruitment

This was a descriptive qualitative study with individual semi‐structured interviews for Finnish registered nurses conducted in March 2021. Participants were recruited from care workers' trade unions within a research project with a survey in which respondents (*n* = 7925) were informed about an opportunity to enrol in interviews. Altogether, 403 care workers volunteered, and of those, 64 were registered nurses. To form a reasonable but sufficient group of participants to answer the research question (Polit & Beck, 2010), we decided to invite all the nurses who were members of one nurses' union (*n* = 34). Finally, 23 nurses (Table 1) were interviewed, including the three pilot test interviews. The amount of participants was assessed to be purposeful due to the saturation, which emerged as an accumulation of participants' repeated parallel perceptions regarding the interview guide themes (Polit & Beck, 2010). We have followed the consolidated criteria for reporting qualitative research (COREQ) checklist (Tong et al., 2007; Table S1) for reporting.

**TABLE 1:** Participants' background information

Age	*n* = 23
29–30	1
31–40 (*n* = 2)^a^	5
41–50 (*n* = 1)^a^	8
51–60	8
60+	1
Work experience in years	*n* = 23
1–10	9
11–20 (*n* = 3)^a^	6
21–30	4
31–40	2
41+	2
Current workplace	*n* = 23
Hospital (*n* = 2)^a^	9
Health centre, home care (*n* = 1)^a^	7
Nursing home	3
Foundation	2
Other than health sector	2
Other profession in health field	*n* = 13
Primary nurse	9
Public health nurse	2
Housekeeper	2

^a^ Included pilot test participants (*N* = 3).

### 2.2 Data collection

We collected data with semi‐structured individual interviews to allow participants broadly describe their perceptions within the topic of nurses' career and calling. We developed the interview guide to provide a uniform skeleton for the interviews (Kallio et al., 2016). Based on previous research (e.g. Carter, 2014), we formulated three main themes with follow‐up questions that focused on nurses' career and calling. We pilot tested the guide with three randomly selected registered nurses. Based on the pilot test, we revised expressions to make the questions more practical and understandable (Table S2). We included pilot interviews in the data due to their valuable contents. Individual pilot and research interviews were conducted by one researcher (M. H. or H. K.) using Zoom videoconferencing software in March 2021. Interviews lasted from 30 to 80 min (52 min on average). The total length of the recordings was 19 h and 50 min. This study reports the career results. Findings focused on calling have been presented by Kallio et al. (2022).

### 2.3 Analysis

The analysis method was data‐driven thematic analysis to provide a rich and diverse description of the data (Braun & Clarke, 2006). Recordings were transcribed, producing 309 pages of text (Calibri 12, single‐spaced). After becoming familiar with the transcripts, the researcher (H. K.) coded and collated the data to sub and main themes using NVivo 12 software (Braun & Clarke, 2006). The initial analysis was discussed, refined and finalized by the research team.

### 2.4 Ethics

Ethical principles were followed throughout the research process (ALLEA, 2017). In Finland, ethics committee approval is not required when adult participants are voluntary and competent (Ministry of Social Affairs and Health, 1999). Permissions for data collection were obtained from the research councils of each trade union and workforce leasing company. We obtained informed consents from the participants, electronically and verbally, and provided them research information, including voluntariness and the right to withdraw (Finnish Advisory Board on Research Integrity, 2012).

## 3 RESULTS

**FIGURE 1:**
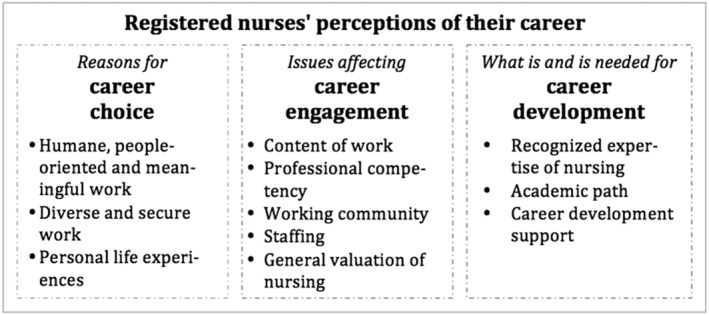
Nurses' perceptions of their career

The results reflect nurses' perceptions of their reasons for choosing nursing as a career and issues having an effect on their career engagement. Results also show what nurses viewed career development to be like and what kind of support was needed for it (Figure 1).

### 3.1 Career choice

#### 3.1.1 Humane, people oriented and meaningful work

Participants had chosen a nursing career, which they considered to be humane and meaningful work. This meant that working with people, helping them and being of benefit to others were elements of work that had been important to them. Participants had also been interested in biology, health, and the hospital world and had felt respect towards the nursing profession and admiration towards its distinctive symbols, such as work clothing. Before deciding on their nursing careers, other pathways emphasized among the participants were related to medicine, pedagogy, theology and languages.

I do not have to think about whether I'm doing meaningful work, because I know I am. (N12)

#### 3.1.2 Diverse and secure work

Diversity and security of work options contributed to nurses' career choice. Participants saw that as nurses they had good opportunities to choose from different workplaces and that nurses' work description is more varied than that of many other professions. They said that nurses are always needed and therefore income is regular.

In nursing, there are a lot of opportunities, so many different specialities that you can apply for, day jobs, shift work, double shift work, entrepreneurship, et cetera. (N22)

If you want to ensure yourself a job that never ends, then this is the right field. (N14)

#### 3.1.3 Personal life experiences

Participants' personal experiences contributed to why they chose nursing careers. They said that some of their loved ones had acted as nurse role models or encouraged them to select nursing. One reason for choosing a nursing career was a crisis, often a serious disease, in their own or a loved one's life. Work as nurses enabled the participants to realize their need to help others or to treat themselves.

I think that it [choosing a nursing profession] was also influenced by things like that one of my grandparents died around that time and another got cancer. (N21)

### 3.2 Career engagement

#### 3.2.1 Content of work

The content of the work had a central influence on nurses' career engagement. Direct patient care was important; nurses found it rewarding to be able to help patients, see them recover and receive positive feedback from them. Nurses also emphasized the importance of abundantly challenging and versatile work, meaning that narrow and repetitive work tasks were insufficient. They also brought up that it is important to work in a field and work environment suitable to one's personal interests. One issue that participants considered to be motivating was development possibilities as part of their work so that the problems they encountered were solved and they could be part of this process.

The maternity ward is such a wonderful ward for me, no matter how busy it is … the same kind of rush, for example, in an internal medicine ward do distresses me. (N22)

#### 3.2.2 Professional competency

Nurses found high professional competency to be important for their career engagement. Experience of managing their own work was important, and being able to handle the wide field of nursing was described as enjoyable. In‐service training was desired, and learning new things was not only needed to answer work demands but also contributed to a meaningful working life experience. In training, one could also find new areas of nursing that would actually interest them. Nurses wanted to apply their professional competency to what it was meant for, and it was crucial that their work tasks corresponded to their training. However, nurses reported that often they are expected to execute non‐nursing tasks suited rather to cleaners, physicians or secretaries.

[In my current work] I have a perfect sense of work management all the time … It's the first time when I really have awakened to think that this is what working as a nurse should be like! (N4)

#### 3.2.3 Working community

Our analysis showed the working community to be central in relation to nurses' experience of a meaningful career. Functional multi‐professional collaboration and networking without hierarchies were important, referring to relationships between nurses and other professional groups and relationships between permanent and temporary employers, as well as collegiality among nurses. Where strong we‐feeling and positive feedback among nurses had empowered them in working life, colleagues' unprofessional attitudes and behaviour had an opposite impact.

This is not a sport of individual performance but team play. It is really important and affects coping at work. (H4)

#### 3.2.4 Leadership

Organizational and managerial work impacted how nurses engaged in their careers. Motivating administrative leadership included respect towards staff, open multilevel dialogue and staff involvement in decision‐making, but nurses also described situations of unfair treatment and exclusion in their organizations. Nurse managers also played a role in nurses' motivation. Participants considered managers' supportive, advocating and inspiring ways of practicing as well as understanding towards their work to motivate them. But they reiterated that also nurse managers need administrative support in their work. Furthermore, employment contract‐related issues were connected to career engagement. In addition to salary, the possibility to choose permanent employment, ergonomic working time planning and opportunities for remote work were such issues.

I have such a good foreman. … It is the managerial work that has made me stay in this [nursing] position. (N22)

#### 3.2.5 Staffing

One crucial factor that affected nurses' career engagement was staffing. Participants said that in general, staffing had been insufficient considering their workload, the number of patients, increased work demands and constant changes, such as digitalization in the health care sector. COVID‐19 had further increased nurses' burden. The need for haste forced nurses to lower the care quality, placing them in ethical conflicts. However, one nurse also mentioned that sometimes her colleagues complain about haste but use their time poorly.

The lack of resources affects daily routine so much that a career doesn't seem meaningful. (N22)

#### 3.2.6 General valuation of nursing

Nurses described the general valuation of nursing and people's understanding of nurses' work to be low, leading them to consider leaving their nursing career. They reported that nurses' low pay was a concrete indication of discrimination against their profession and that people's negative comments in social media about nurses reflect their incomprehension of nursing work. According to their experiences, people expect nurses to have a humble character, be content with their working conditions and never express criticism. On the other hand, participants said that nurses often lack the courage to defend themselves. Nurses considered trade unions' advocacy activities vital for working life improvements.

Those who are supposed to appreciate nurses, employers and governments, don't value a bit … That eats nurses’ motivation to do this work. (N19)

### 3.3 Career development

#### 3.3.1 Career development as recognized expertise of nursing

Participants saw career development in nursing as guiding their career to refine their area of expertise and increase their competency. Expertise could be focusing on a certain narrow area of nursing, such as stoma care or resuscitation, or a wider specialty, such as cardiac or palliative care. In this kind of role, a nurse who works with patients could delve into the area of the professional competency, perform special tasks and share knowledge in the working community. Nurses also connected increased expertise with opportunities to work independently and have greater influence on their work and organizational issues. According to them, career development needed to be recognized and correlate with wages because of the expansion of competency and responsibilities.

You kind of rise from a regular nurse to a development task or something like that. (N17)

Perhaps, it [career development] is deepening of professionalism. (N12)

#### 3.3.2 Career development as academic path

When discussing nurses' options for career development, participants brought up the possibility of proceeding into an academic nursing position, which required acquiring a new degree. A degree from a university of applied sciences was required for clinical specialist's work, although team leaders or responsible nurses in the patient care units often had this training as well. Nurses said that proceeding on leadership stairs is a ‘traditional’ image of nursing career development. The leadership path, as well as working as a nursing teacher or researcher, required university training. However, participants underlined that these roles were outside of the nurse title and did not consider them as actual development in the nursing career itself. Regarding the career development options, project, association and workplace steward roles were also brought up, but these kinds of tasks were temporary and rare.

There is an opportunity to study further from a nurse, to be assistant head nurse or nurse manager, but basically there is no way to progress anywhere within this [nurse] role. (N5)

#### 3.3.3 Career development support

Participants brought up different support mechanisms that nurses need to be able to develop their career in patient care. Participants perceived employers' supportive attitude and actions to be vital: employers positively relating on career development, making development options visible in the organization and enabling nurses' studies during working time and at the employer's expense. Support could also manifest as encouraging nurses to apply to new positions, empowering them in their new roles and advocating their rewarding in organizations. Colleagues' attitudes were also connected to career development. Participants called for working communities in which nurses respected each other's competence and development. However, individual career pursuits also required a combination of personal will, courage and motivation for a change in working life, as well as a suitable life situation for studying.

In my opinion, nurse's career development is dependent on employer's investments in these issues. (N18)

My supervisor said she would request a higher personal bonus for me because I have taken on this new area of responsibility. (N14)

## 4 DISCUSSION

Based on this study, a nursing career with direct patient care was desirable. Nurses had chosen their career because of the people oriented and humane nature of nursing, the element that appears to be persistent over time (Carter, 2014; Eley et al., 2012). Throughout their working lives, patient care and practice tended to remain crucial for nurses' experience of a meaningful career. However, nurses perceived career opportunities, models and support within direct patient care to be lacking. Instead, they connected career development in nursing to moving away from patients. In addition, they experienced a lack of societal support for and valuation of a nursing career.

Based on our results, work structure and environment‐related issues also had an impact on nurses' careers. Structures were particularly emphasized regarding insufficient staffing resources (e.g. Marufu et al., 2021). Nurses missed permanent colleagues and contracts. Permanence not only increased one's security in a career and thus in life but also impacted on colleagues' work because the use of substitute workers increases permanent ones' responsibilities. Regarding their work environment, constant reformations burdened nurses and hindered their experience of a meaningful career. One factor was technology, as constant learning of new programmes burdened nurses, taking energy and time from other learning and development work.

It is worth noticing that it may be beneficial in the longer term to also encourage those nurses who are uninterested in career development because they want to stay in their current role. Research has shown that in the longer term, extending the boundaries of practice may lead to increased autonomy and responsibilities and promote confidence and empowerment (Rasmussen et al., 2018) as well as engagement (Arrowsmith et al., 2016). Overall, professional advancement activities have been proven to promote retention of nursing staff and reduce turnover (Brook et al., 2019); however, activities are not widely used for these purposes, as our results showed.

Nurses in this study considered nursing as a career to be somewhat dull. Nurses expressed their interest towards career development and connected it to meaningful work but saw their opportunities for it as scarce, even non‐existent. If a nurse were to proceed in a career, it meant abandoning direct patient care, the element that made them to choose the career and gave its meaning (Karlsson et al., 2019). Weak career development possibilities may lead to decreased job satisfaction and leaving the profession (Zhu et al., 2021). Therefore, health care organizations should develop career paths that allow patient care practice throughout the career to that could prevent nurses from being frustrated and leaving the profession and instead foster professional identity (Rasmussen et al., 2018).

Another challenge in career advancement in nursing is that employers had supported nurses' career development very little (also Price & Reichert, 2017). Our study showed that nurses had trouble, describing what career development in nursing could be and that they recognized only minor opportunities for it. In line with previous research, our results thus emphasize the need for nurses' career mentoring (Tucker & Gallagher‐Ford, 2019) and nurse managers' role in strengthening nurses' career planning and introducing them possible career goals already at the beginning of their careers (Yan et al., 2021). Some leadership styles have been shown to be beneficial to nurses' careers and are therefore worth taking into account in nurse managers' degree programmes and continuing education. For example, an authentic leadership style has been shown to increase nurses' career satisfaction, decrease career turnover intentions (Alilyyani et al., 2018) and servant leadership professional development (James et al., 2021).

Nurse managers, but also nurses themselves from the very beginning of their nursing career, should be aware of career development opportunities and possibilities, because a successful career needs planning and networking (Carter, 2014). Thus, scrutinizing nurses' career development thoughts, wishes and ideas and opportunities to answer them is an important part of development discussions and regular updates between an employee and a supervisor and degree education in nursing. The nurse managers' role is also central in making the organization's career path models familiar to nurses (Moore et al., 2019).

Lack of career development programmes also seems to be problematic, considering the quality of patient care. Previous studies have brought up that, as career development is connected to professional competency improvement, it is likely to extend professional values, such as promoting care quality and patient safety (Haines et al., 2021; Yarbrough et al., 2017). In this study, an even salary for all the nurses regardless of their work performance was brought up. Those who aimed for high quality in their work were not rewarded. This is prone to causing resentment but also to hinder motivation to perform the best.

Poorly conceived career development options might be one of the reasons why nursing mainly remains an undervalued women's profession with low pay and poor working conditions (Bayliss‐Pratt et al., 2020). These adverse working conditions, along with underdeveloped career advancement options, lack of in‐service training (Simpson & Simpson, 2019) and also nursing's inherent challenges (Kox et al., 2020) may change nursing into a job without security (Bodin et al., 2020). Precarious working conditions, unfortunately, already hamper the quality of working life in nursing, manifested, in addition to the above‐mentioned issues, as low autonomy, poor control over working times, overwork and high demands (Bayliss‐Pratt et al., 2020; Hult et al., 2022). Like the participants in this study, many nurses currently intend to leave nursing, though the lack of career development options is only one reason among others.

### 4.1 Limitations

We interviewed nurses during the COVID‐19 pandemic. Considering motivational and career development issues in this kind of burdensome situation may be secondary for them and thus produce biased findings compared with a more conventional situation. Also, the COVID‐19 pandemic has been shown to increase nurses' dissatisfaction with work and intentions to leave (Lavoie‐Tremblay et al., 2022). On the other hand, data collection with remote access in the time of the pandemic enabled nurses to participate at times convenient for them. We did not carry out repeat interviews or return transcripts and request feedback.

## 5 CONCLUSIONS

Traditional perceptions of caring professions still seem to attract people to nursing careers and engage them. Reasonable challenges and development contribute to a meaningful working life, and career development opportunities could increase the attractiveness and general valuation of nursing. Based on our study, a level of nursing that combines patient care, continuous competence and practice development with a clear reward system is desired among nurses. As the greatest part of nursing professionals works in direct patient care, career development within that sector is a central question for ensuring the nursing workforce of the future. Pay and organizational involvement are also critical in increasing nurses' motivation to stay, and they need to be further developed. In the future, nurses' career development models should be studied in relation to the content and diversity of their work.

## 6 IMPLICATIONS FOR NURSING MANAGEMENT

Nurse leaders have a significant role in supporting nurses' motivation and engagement in their careers. This study showed that nurses value increased competency and development possibilities, which lead them to wish for reasonably challenging work tasks. Nurse managers' support and encouragement and, concretely, allocation of working time for training are some of the factors that may increase the meaningfulness and motivation for nurses to engage in their career. Nurses need clear, equal and motivating career paths and models; however, nurses who want to do a very basic job should also be supported. This study showed that career advancement should also be reflected in pay. Nevertheless, the study found that advocacy and involvement in decision‐making are as important as pay. Nurse managers and leaders should foster involvement and interest in career development by applying a respectful and inspiring leadership style.

## CONFLICTS OF INTEREST

The authors have no conflicts of interest to declare.

## ETHICS STATEMENT

Ethical principles were followed throughout the research process (ALLEA, 2017). In Finland, ethics committee approval is not required for this type of study, which interviews adults who volunteer and are competent (Ministry of Social Affairs and Health, 1999). However, permissions for data collection were obtained from the research councils of each trade union and workforce leasing company before the data collection phase. We obtained informed consents from the participants, electronically and verbally, and provided them research information, including voluntariness and the right to withdraw (Finnish Advisory Board on Research Integrity, 2012).

## Supporting information


**Table S1.** Nursing career; COREQ‐checklist.

**Table S2.** The semi‐structured interview guide.

## Data Availability

Research data are not shared.
