# Supplementary material for: Registered nurses' perceptions of their career—An interview study
Source: J Nurs Manag. 2022 Sep 16;30(7):3378–85. doi: 10.1111/jonm.13796 (PMC10087756; doi:10.1111/jonm.13796)
Supplement: Supplementary file 1 — Table S1. Nursing career; COREQ‐checklist. [file JONM-30-3378-s002.docx]

Supplementary file 1.

Nursing career; COREQ-checklist.

| **No** | **Item to be checked** | **Section of the text and description** |
| --- | --- | --- |
| Domain 1. Research team and reflexivity | | |
| 1 | Facilitators | Data collection |
| 2 | Credentials | Title page |
| 3 | Occupation | Tile page |
| 4 | Gender | Title page |
| 5 | Experience and training | Data collection |
| 6 | Relationship established | Study design, participants and recruitment |
| 7 | Participant knowledge of the interviewer | Data collection |
| 8 | Facilitator characteristics | Title page |
| Domain 2. Study design | | |
| 9 | Methodological orientation and theory | Study design, participants and recruitment |
| 10 | Sampling | Study design, participants and recruitment |
| 11 | Method of approach | Study design, participants and recruitment |
| 12 | Sample size | Study design, participants and recruitment |
| 13 | Non-participation | Study design, participants and recruitment |
| 14 | Setting of data collection | Data collection |
| 15 | Presence of non-participants | Not applicable |
| 16 | Description of sample | Study design, participants and recruitment |
| 17 | Data collection instrument | Data collection, Supplementary file 2 |
| 18 | Repeat interviews | Limitations |
| 19 | Audio recording | Data collection |
| 20 | Field notes | Not applicable |
| 21 | Duration | Data collection |
| 22 | Data saturation | Limitations |
| 23 | Transcripts returned | Limitations |
| Domain 3. Analysis and findings | | |
| 24 | Number of data coders | Analysis |
| 25 | Description of the coding tree | (see 31, 32 below) |
| 26 | Derivation of themes | Analysis |
| 27 | Software | Analysis |
| 28 | Participant checklist | Not applicable |
| 29 | Quotations | Results |
| 30 | Data and findings consistent | Findings |
| 31 | Clarity of major themes | Findings (headings) |
| 32 | Clarity of minor themes | Findings (sub-headings) |
